# Exosomes in the tumor microenvironment as mediators of cancer therapy resistance

**DOI:** 10.1186/s12943-019-0975-5

**Published:** 2019-03-01

**Authors:** Irene Li, Barzin Y. Nabet

**Affiliations:** 1 Stanford Cancer Biology Program, Stanford University, 318 Campus Drive, Stanford, CA 94305 USA; 2 Department of Radiology, Stanford University, 318 Campus Drive, Stanford, CA 94305 USA; 3 Department of Radiation Oncology, Stanford University, 265 Campus Drive, Stanford, CA 94305 USA; 4 Stanford Cancer Institute, Stanford University, 265 Campus Drive, Stanford, CA 94305 USA

**Keywords:** Exosomes, Tumor microenvironment, Therapy resistance, Biomarkers

## Abstract

Exosomes are small extracellular vesicles that contain genetic material, proteins, and lipids. They function as potent signaling molecules between cancer cells and the surrounding cells that comprise the tumor microenvironment (TME). Exosomes derived from both tumor and stromal cells have been implicated in all stages of cancer progression and play an important role in therapy resistance. Moreover, due to their nature as mediators of cell-cell communication, they are integral to TME-dependent therapy resistance. In this review, we discuss current exosome isolation and profiling techniques and their role in TME interactions and therapy resistance. We also explore emerging clinical applications of both exosomes as biomarkers, direct therapeutic targets, and engineered nanocarriers. In order to fully understand the TME, careful interrogation of exosomes and their cargo is critical. This understanding is a promising avenue for the development of effective clinical applications.

## Background

The tumor microenvironment (TME) is a complex ecosystem and an active participant in all stages of cancer initiation and progression [1, 2]. Comprised of diverse cell types in a variety of functional niches, the TME modulates a plethora of cell-cell interactions. These interactions orchestrate reprogramming into cancer-permissive environments and can have significant impacts on cancer development [3], progression [4], and treatment success [5]. Therapies targeting the immune compartment of the TME are promising, especially in combinatorial approaches. However, the TME has been implicated as a major source of therapy resistance, especially due to its inherent heterogeneity and adaptability [6]. With the advent of single-cell technologies, cancer heterogeneity has been interrogated in cancer cells [7, 8] and the surrounding TME [9–11]. This heterogeneity is complicated by dynamic signaling. Cells in the TME exchange information through a variety of signaling networks, ranging from juxtacrine interactions such as desmosomes and cell-cell junctions, to secreted factors such as cytokines, chemokines, and extracellular vesicles such as exosomes [12]. Exosomes and other extracellular vesicles highlight the complexity of dynamic cell-to-cell interactions that make up the TME.

In this review, we focus on our growing understanding of the biogenesis and functions of exosomes originating from cancer cells and the TME and their ability to mediate paracrine signaling and influence cancer progression. In total, the TME can amplify critical oncogenic pathways in cancer cells to promote tumor progression, dissemination, and therapy resistance. Exosomes are an intriguing component of TME signaling and represent a growing body of research that may lead to exciting clinical applications and therapies.

## Characteristics of exosomes

### Exosome biogenesis

Exosomes are small (< 150 nm), extracellular vesicles that form by a dynamic endocytic process [13]. In the process of endosomal maturation, intraluminal vesicles form via ESCRT-dependent and independent processes [14]. The dual invagination at the plasma membrane to form endosomes and subsequent intraluminal vesicles results in a double-layered lipid membrane in the same orientation as the originating cell’s plasma membrane. This orientation and structure are crucial to exosomes’ ability to efficiently mediate cell-cell interactions. The late intraluminal vesicle-containing endosomes are referred to as multivesicular endosomes or multivesicular bodies. The contents of the intraluminal vesicles that become exosomes contain directly sorted and sometimes stochastically acquired cytoplasmic and membrane-bound contents. Generally, multivesicular bodies will fuse with lysosomes to degrade or recycle their contexts. Extracellular vesicles released from multivesicular bodies that fuse with the plasma membrane are known as exosomes [13, 14]. These are not to be confused with microvesicles, which form by budding from the plasma membrane. The term exosome is often incorrectly used interchangeably with extracellular vesicle. In contrast, exosomes are a subset of extracellular vesicles that originate from endosomes and are difficult to distinguish from other small extracellular vesicles by common isolation methods. While this does not detract from previous findings on exosomes, it is important to note.

### Exosome isolation techniques

**Fig. 1:**
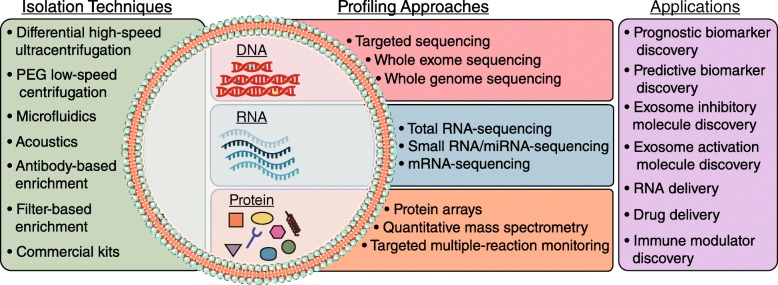
Exosome isolation techniques, contents, and applications. Standard and emerging exosome isolation techniques, general overview of exosomal contents, the techniques to profile these contents, and applications of exosome study

Exosomes can be isolated from cell culture supernatants and biological fluids using a variety of techniques (Fig. 1). In tissue culture models, exosomes are classically separated from input media by differential high-speed ultracentrifugation, including steps to clear cells, cell debris, and larger microvesicles [15]. While this technique is most prevalent, it can result in inconsistent yield and purity, and the harsh nature of ultracentrifugation can destroy exosomes [15]. Another isolation technique is polyethylene glycol-based low-speed centrifugation – however, it is unclear if this method interferes with the functionality of purified exosomes [16]. Several commercial exosome isolation kits are widely used, but their ability to yield pure and functional exosomes is poorly characterized. Further, antibody- and filter-based enrichment methods can produce pure populations of exosomes without harsh centrifugation [17]. More recently, methods that incorporate acoustics and/or microfluidics have been developed. These methods separate exosomes from cell culture or biological fluids in a label-free and contact-free manner [18, 19]. Acoustofluidic approaches use acoustic waves in the context of a microfluidic device to perform size-based separation from whole blood [19]. Fluidic techniques, such as ExoTIC (exosome total isolation chip) use a step-wise nanoporous membrane approach to enrich and then further purify extracellular vesicles in the 30-200 nm range [18]. Once widely available, acoustofluidic or fluidic methods may be the most accurate approaches to isolate reproducible quantities of functional and intact exosomes.

There are also several necessary considerations for isolation of exosomes or extracellular vesicles from tissues. Exosomes can be isolated from the conditioned medium of ex vivo cultured tissues, or directly from tissues. In the case of direct isolation from whole tissue, it is imperative to use gentle dissociation of the tissue to minimize disruption of cell integrity, which may result in cellular vesicle contamination [20]. Isolation from the conditioned medium of ex vivo cultured tissues should result in a more pure exosome population; however, the nature of ex vivo culture may result in contents that may not exactly reflect native contents able to be isolated from whole tissue [20].

After isolation, exosomes must be quantified and assessed for purity. Due to their small size, quantification is not trivial. A rough estimate of quantity is possible by protein quantification, but direct quantification is best done by nanoparticle tracking analysis using technology such as NanoSight. These techniques use light scattering and Brownian motion to accurately identify the size and quantity of exosomes in a suspension [21]. Flow cytometry can also indirectly quantify exosomes by binding exosomes to larger beads [21]. Purity and quality of exosomes is best assessed by electron microscopy, where the classical “cup-like” structure and lipid bilayer should be observed [15]. Electron microscopy can also identify evidence of exosome destruction or macromolecular structures that may result from high-speed ultracentrifugation. Exosome purity can also be assessed by the presence or absence of protein markers. Because exosomes can contain a snapshot of the cell of origin, many proteins present in cells will be present in exosomes to some degree. Generally, probing for the presence of exosome structural molecules, such as the tetraspanins TSG101, CD81, and CD9, and an absence of histone proteins can confirm a lack of cellular contamination [20]. Whenever possible, all the above confirmations should be completed to assure reproducible results.

### Exosome contents

As mentioned, exosomes contain molecules also found in their cell of origin, potentially through a targeted sorting mechanism (Fig. 1). Due to their biogenesis from endocytic pathways, exosomes can be defined by endocytic proteins. They also contain cell-type specific exosomal proteins largely of cytoplasmic origin, including adhesion molecules, cytoskeletal proteins, enzymes, and other transmembrane proteins [13]. Lipids are also a key component of exosomes. Specifically, sphingomyelin, phosphatidylserine, cholesterol, and saturated fatty acids have been demonstrated to be enriched in exosomes, compared to cells [22]. Collectively, the enrichment of specific lipid and protein contents in exosomes suggests a targeted cellular sorting mechanism.

Exosomes also contain nucleic acids: genomic and mitochondrial DNA have been reported in exosomes, but the varieties of exosomal RNA species are best characterized [23–25] (Fig. 1). Exosomal RNA differs from cellular RNA in that it is largely bereft of full-length ribosomal RNA (rRNA) that makes up more than 95% of the human transcriptome [25]. Functional mRNAs are present in exosomes, but they comprise a small fraction of total exosomal RNA contents. Exosomes largely contain non-coding RNA (ncRNA), including microRNA. These RNAs are resistant to RNase digestion, suggesting they are within exosomes, rather than exposed on the cell surface. Moreover, the advent of next-generation sequencing (NGS) technologies has allowed for an explosion of reports of exosomal RNA contents, but few unifying properties other than a general enrichment for ncRNA have been identified to date. Thus, the functional genomic content of exosomes is context-specific and is continually being uncovered.

### Emerging areas of exosomal study

Historically, exosome protein content from different cell types and biological fluids has been characterized by low-content approaches such as Western blotting and flow cytometry. More recently, mass spectrometry-based analyses have increased the depth and breadth of proteomic profiling of exosomes. While the protein profile of exosomes largely resembles the cell they were derived from, quantitative mass spectrometry approaches are able to differentiate this with higher resolution [26]. Beyond differential protein expression analyses, quantitative mass spectrometry can reliably differentiate post-translational modifications that may be enriched in exosomes. High-content proteomic profiling will not only inform exosome biology and function, but also enable improved isolation and characterization of exosomes (Fig. 1).

The advent of next-generation sequencing technologies has resulted in an explosion of data, and exosomes are no exception. Exosomes from cell culture and biological fluids have been subjected to both RNA and DNA sequencing to understand their contents and function. In particular, RNA-seq of exosomal contents has demonstrated few unifying theories besides a lack of ribosomal RNA and enrichment of ncRNA. Depending on the RNA-seq approach, miRNAs may be enriched or depleted, resulting in little overlap between published studies. To address some of these challenges and standardize methods, the NIH Common Fund launched the Extracellular RNA Communication Consortium (ERCC) in 2013 with the goal of understanding extracellular RNA secretion, delivery, and function in recipient cells [27]. The ERCC also aims to describe extracellular RNA species in human biofluids, test the clinical utility of these RNAs, and provide a data repository for these studies. This effort has resulted in several databases such as Vesiclepedia, exRNA, and ExoCarta, to query not only exosomal RNA-seq data but also DNA and protein arrays [28]. By providing a repository of data, protocols, and high-quality publications, the fundamentals of exosome biology may be more rigorously uncovered (Fig. 1).

## Role and function in Cancer

### Homotypic exosome transfer between cancer cells

Due to their content and signaling capacity, exosomes have been implicated in a host of processes related to the progression of various cancer types. Exosomes participate in signaling from cancer cells to other cancer cells to propagate cell growth, transformation, and survival signals. In glioblastoma, exosomes were shown to transfer functional EGFRvIII protein, aiding in the transformation of wildtype cells [29]. Similarly, exosomes from patients and breast cancer cell lines were demonstrated to contain miRNA processing machinery and deliver miRNAs that induced transformation and tumor formation in non-tumorigenic mammary cells [30]. In established tumors, glioma-derived exosomes can transfer functional mRNAs and miRNAs that promote tumor growth [31, 32]. Further, autocrine signaling via cancer cell-derived exosomes can provide strong progression signals. For example, exosomes isolated from gastric cancer cells in vitro promoted proliferation in an Akt/PI3K and MAP kinase signaling-dependent manner [33]. Similarly, pancreatic cancer exosomes encourage cancer survival by modifying signaling via the Notch-1 signaling pathway [34]. In total, cancer cells can utilize exosomes in homotypic transfer to enable cancer progression via oncogenic pathways.

### Heterotypic exosome transfer in the TME

**Fig. 2:**
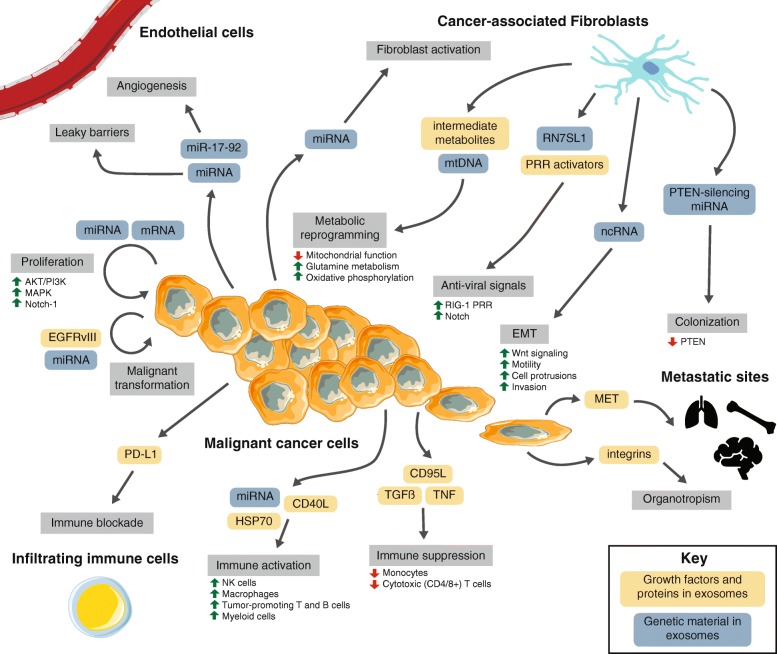
Tumor microenvironment interactions. A macroscopic view of the molecular crosstalk between cancer-associated fibroblasts, endothelial vasculature, infiltrating immune cells, and malignant cells in the TME. Dynamic interactions governed by heterotypic signaling mechanisms between cell types modulate various stages of cancer progression (grey boxes). The role of exosomes in this cell-cell signaling is highlighted (blue and orange boxes)

The growth, progression, and dissemination of a tumor is supported by its local tumor microenvironment (TME), a system of diverse cell types that participates in all stages of cancer initiation and progression. Essential hallmarks of cancer, such as sustaining proliferation, evading growth suppression, avoiding immune recognition, activating invasion and metastatic cascades, resisting cell death, initiating angiogenesis, and deregulating cellular energetics, are influenced by the tumor microenvironment [1, 2]. The TME modulates many cell-cell interactions through a variety of signaling networks, including juxtacrine and paracrine interactions [12]. Of the paracrine signaling interactions, exosomes are an important and emerging mechanism of cell-cell communication (Fig. 2).

The TME comprises a multitude of cell types that can be broadly organized into endothelial, fibroblastic, and immune cells. These cells are surrounded by and suspended in the extracellular matrix. Each cell type is heterogeneous and contains multiple subtypes and classifications. Commensurate with this complexity, they participate in diverse signaling interactions that vary extensively by organ, cancer type, and patient. These interactions not only support cancer growth, but also may be an essential component of cancer cells’ ability to grow and adapt when challenged with therapies. Understanding these signaling networks, especially exosomal contributions, may identify potential targets to combat therapy resistance (Fig. 2).

#### Endothelial cells

Endothelial cells form vasculature, which funnel nutrients and waste products from the tumor core – this is necessary for tumors to grow and invade past the diffusion limit of oxygen. Crosstalk between endothelial and malignant cells stimulates growth in both cell types and sustains therapy resistance [35, 36]. Malignant cells and some subsets of immune cells can encourage initiation of angiogenesis by expression and secretion of growth factors such as VEGF, TNF, and MCP-1 and induction of hypoxia [37, 38], which can cause leaky vessel structures that encourage metastatic dissemination. Recent studies observe that exosome-mediated miRNA transfer from cancer cells to endothelial cells aids in the destruction of endothelial cell barriers and cancer cell release into the blood stream for dissemination and metastasis [39]. Further, leukemia cells secrete miRNA-containing exosomes – mainly of the miR-17-92 cluster – which can induce endothelial cell migration and maturation, typical of cancer angiogenesis [40].

#### Fibroblasts

The fibroblasts associated with cancerous lesions are a dominant compartment of the TME and denoted as cancer-associated fibroblasts (CAFs) [41]. Fibroblasts are well-suited to actively support cancer cells due to their stress resistance, plasticity, participation in signaling and cell-cell interactions, and function in wound healing and fibrosis and have both tumor-promoting and tumor-restrictive impacts [42]. On one hand, fibroblasts promote tumorigenesis by influencing the microenvironmental secretome, which sustains inflammation [43], modulates immune recruitment [44], sustains CAF activation [45], and directly engages cancer cells to sustain tumor proliferation and enhance invasion and metastasis [46]. Additionally, CAFs promote cancer invasion by producing matrix metalloproteinases that reshape the extracellular matrix of the TME [47] and intensify hypoxic conditions [48]. Conversely, specific subsets of fibroblasts have been shown to directly oppose these mechanisms and combat tumor growth [49, 50].

Exosomes are a crucial component of heterotypic fibroblast and cancer cell signaling. For example, exosomes secreted by leukemia cells have been shown to accelerate CAF activation to remodeling the TME and extracellular matrix to a more cancer-permissive state [51]. Moreover, fibroblast exosomes have been demonstrated to enhance the migratory capacity of breast cancer cells by activating Wnt-signaling pathways [52]. Additionally, exosomes from prostate CAFs have been observed to encourage TME metabolism toward a glycolytic, less oxidative profile typical of solid cancers by downregulating mitochondrial function, enhancing glutamine metabolism, and serving as a source of intermediate metabolites [53]. Fibroblast-derived exosomes have also been shown to contain mtDNA that activates oxidative phosphorylation in recipient breast cancer cells, leading to endocrine therapy resistance [54]. Another study indicated that exosomes from CAFs prime drug resistance mechanisms in colorectal cancer stem cells, accelerating drug resistance via paracrine signaling [55].

In basal-like and triple-negative breast cancer, we uncovered a complex heterotypic signaling cascade between stromal fibroblasts and cancer cells [56]. Fibroblasts secrete ncRNA-containing exosomes, which upregulate anti-viral signaling in recipient breast cancer cells by activating the RIG-I pattern recognition receptor (PRR) [57]. This directly coordinates with parallel activation of Notch signaling in the breast cancer cells, ultimately enriching for cells that are adept at both tumor initiation and resistance to conventional chemo/radiotherapy [56]. In a follow-up to this work, we identified one stromal RNA transcript, RN7SL1, that is recognized by and activates RIG-I. Mechanistically, we demonstrated that when stromal fibroblasts and basal-like breast cancer cells interact, fibroblast-derived exosomal RN7SL1 is not bound by its canonical RBPs and can act as a potent activator of RIG-I in recipient breast cancer cells. This is due to a transcriptional upregulation of RN7SL1 RNA, while the RBPs that normally bind it do not concomitantly increase. Therefore, the balance of RNA to RBP is tipped in favor of excess RNA. Expanding these findings to human patients, we demonstrated that the serum of triple-negative breast cancer patients is enriched for unshielded RN7SL1, whereas serum from healthy individuals and patients with other breast cancer subtypes is not [57]. In total, exosome-mediated interactions between CAFs and cancer cells can regulate various cancer promoting pathways and we expect that careful examination of these heterotypic interactions and downstream targets may reveal additional tumor-promoting signaling cascades.

#### Immune *cells*

Immune cells in the TME secrete chemokines, cytokines, growth factors, and proteolytic enzymes, which can encourage tumor progression, modulate immune evasion, or actively kill tumor cells [58]. Further, the recruitment and migration of immune cells into the tumor microenvironment is governed by dynamic signaling [59], and exosomes are a key component of these interactions [60]. Exosomes from cancer cells may directly activate natural killer (NK) cells, macrophages, B cells, and T cells [61]. Recently, it was also demonstrated that activation of canonically oncogenic signals can result in the release of immune-activating exosomes that result in robust tumor clearance [62]. Exosomes can also be immune-suppressive: they may inhibit the cytotoxic activity of effector CD4 and CD8 T cells and NK cells [61]. They can also inhibit differentiation of DCs and myeloid-derived suppressor cells (MDSCs) [63, 64]. A key to understanding exosome-immune signaling may be RNA: we demonstrated that exosomal RNA can activate myeloid cell populations [57]. However, the role of RNA-sensing pathways in the activation or suppression of the innate and adaptive immune system is understudied. Others have demonstrated that miRNAs can function as toll-like receptor (TLR) ligands in several cancers to promote cancer progression [65, 66]. The mechanisms by which exosomes modulate the immune system are varied and continually uncovered, but it is clear that they exert an influence, and that exosomal RNA can function as potent signaling molecules.

A hint to the role of exosomal RNA can be found in the propagation of anti-viral signals and amplification of anti-viral responses. Secretion and transfer of exosomes to uninfected bystander cells can result in exosome-transferred viral RNA by PRRs. For example, cells infected with a Hepatitis C virus (HCV) strain incapable of producing virions can transfer HCV genomic RNA to uninfected cells via exosomes. This HCV RNA is then recognized as a pathogen-associated molecular pattern by recipient cells and anti-viral signaling is activated in the absence of direct virus infection [67]. Similarly, adenoviruses can cause an increase in exosome transfer containing PRR-activating cargo that results in anti-viral signaling and a short-range anti-viral response [68]. In the case of latent Epstein-Barr virus (EBV) infection, exosomal transfer of EBV RNA can alert neighboring cells of an infection. Here, latent-infected cells can trigger an anti-viral response in neighboring cells by the transfer of EBV 5’ppp RNA bereft of any shielding RNA-binding proteins [69]. Together, these studies demonstrate that exosomes can mediate anti-viral signaling within uninfected cells and tissue-level amplification of the anti-viral response. While these studies provide strong evidence for the role of exosomal RNA in the dissemination of anti-viral responses, it is unclear whether activation of RNA recognition pathways in the TME would activate or suppress anti-tumor immune responses and requires further investigation.

### Exosomes in pre-metastatic and metastatic niches

Exosomes play a prominent role in preparing certain organs as pre-metastatic niches, favorable places for future dissemination and metastatic seeding [70, 71]. In melanoma, pre-metastatic niche formation is governed by exosomal transfer of MET to bone marrow progenitor cells, thus encouraging lung metastases [72]. Pancreatic cancer cell-derived exosomes can induce hepatic stellate cells to secrete TGF-β and recruit specific macrophage populations, establishing pre-metastatic niche in the liver [73]. Similar to fibroblasts, astrocytes in the brain metastatic microenvironment can secrete exosomes containing miRNAs that specifically silence PTEN and result in metastatic colonization [74]. Also, exosomes from cancer cells with defined metastatic organotropism contain specific integrins that determine their organotropism [75]. Treatments can also change the content and function of exosomes as they relate to the pre-metastatic niche. It was recently demonstrated that chemotherapy-elicited extracellular vesicles in breast cancer cells can promote pre-metastatic niche formation in the lung by transferring annexin 6 to induce *Ccl2*, which enforces monocyte activation [76]. In total, cancer cell-derived exosomes can have short and long-range effects on other cancer cells or host cells to aid in all steps of cancer progression. Better understanding of these effects will allow the development of critical exosome-informed therapies that overcome therapy resistance.

## Clinical implementation of exosomes

Due to their versatility, exosomes represent a tantalizing target for clinical implementation as engineered nanocarriers for biological compounds and biomarkers of patient disease status. Advances in our understanding and engineering of exosomes, from their molecular characteristics and physiological function, will increase the effectiveness and efficiency of exosome-derived therapies. Given the prevalence of exosome signaling in the TME, clinical methods centered on exosomes may also be a promising avenue for subverting the development of cancer therapy resistance.

### Exosomes in immunotherapy

Antibody-based blockade of immune checkpoints such as CTLA4 and the PD-1/PD-L1 signaling axis have resulted in remarkable and durable responses in various cancers [77]. Unfortunately, the majority of patients do not respond to these therapies alone due to adaptive and acquired resistance mechanisms [78]. There is considerable interest in the combination of immunotherapies with targeted or conventional cytotoxic therapies for the treatment of solid cancers [79]. Understanding the immune-suppressive or -activating role of exosomes present in the tumor microenvironment can ultimately lead to identification of exosome-based biomarkers of response and also to the design of rational combinatorial therapies. Recently, it has been demonstrated that cancer-derived exosomes transfer functional PD-L1 and inhibit immune responses [80]. Further, in melanoma patients receiving PD-1 blockade, exosomal PD-L1 levels correlated with tumor burden and response to therapy. It is unclear whether exosomal PD-L1 directly correlates with tumor or immune PD-L1 status, but it may have utility as a predictive biomarker for PD-1 blockade. Similar to exosomes in conventional therapy, PD-L1-containing exosomes may be both regulators and biomarkers of therapy resistance.

### Exosomes as biomarkers

As more is understood about the fundamentals of exosome biology and how they relate to cancer and cancer therapy resistance, exosomes and the TME are increasingly interesting targets for clinical application. First, exosomes are promising, sensitive, and specific biomarkers of disease, therapy resistance, and treatment response. Because exosomes can come from any cell type in the tumor, they can provide a snapshot of the entire tumor (Fig. 1). As more high-throughput, high-content interrogation of purified exosomes is performed, exosome profiling from a heterogeneous cell population, such as a tumor, could allow future deconvolution of cell types and status in a non-invasive manner.

More currently feasible approaches build on our current understanding of exosomes and the TME. It is thought that more exosomes are produced by more advanced cancers. Therefore, it has been suggested that total circulating exosome burden can identify disease [72]. Exosomal contents can also identify disease. In pancreatic cancer, exosomes isolated from the bloodstream of patients with precancerous lesions or pancreatic cancer contain a membrane bound protein, GPC1, which was demonstrated to be a very sensitive and specific marker of early-stage disease [81]. We showed that certain RNA species are enriched in the sera of patients with triple-negative breast cancer when compared to hormone receptor positive breast cancers [57]. As described above, exosomal PD-L1 may also be a regulator and biomarker of response to PD-1 blockade in melanoma [80]. Endothelial cell-derived exosome content can reflect transient cellular stress conditions and could be useful as indicators of anti-angiogenic therapy effectiveness and cancer cell status [82]. Exosomes may also contain cancer-derived nuclear DNA that can be assessed for mutational status [24, 81]. It is unclear what proportion, if any, of circulating tumor DNA is packaged in exosomes or other extracellular vesicles. It is also important to consider that some current methods of circulating tumor DNA detection are highly sensitive and specific, and exosome isolation may not provide any advantage [83, 84]. Careful characterization of which cell-free compartments contain circulating tumor DNA has not been performed; therefore, the added utility of exosomes for DNA biomarkers is cannot be evaluated. While exosomes have significant promise as biomarkers in cancer, it is crucial that isolation and characterization methods be standardized for clinical implementation. It is likely that improvements in acoustic and/or microfluidic approaches will provide an important step forward in delivering reproducible exosome yield and purity for clinical applications.

### Exosomes as delivery modules

Another approach to clinical implementation of exosomes is as biologically active carriers, providing a platform for enhanced delivery of cargo in vivo. Due to exosomes’ intricate structure, engineering them to be effective and safe requires thorough understanding of their necessary components, including, but not limited to, membrane stability, architecture, organization, and packaging of the interior components [85]. Using biologically derived exosomes as a starting point, groups have worked to re-engineer exosomes to contain small molecule inhibitors, functional genomic material, reporter systems, and targeting peptides, among many others [86, 87]. In all of these cases, toxicity and immunogenicity concerns should be taken into consideration – it has been demonstrated that exosomes derived from stromal cells, such as fibroblasts and DCs, may be effective [88]. In one study, fibroblast-derived exosomes with high CD47 expression were engineered to carry short interfering or short hairpin RNA specific to oncogenic *Kras*^*G12D*^ expression. In mouse models of pancreatic cancer, these engineered exosomes ablated oncogenic KRAS signaling, slowed tumor growth, and increased overall survival [89]. Other studies have also demonstrated exosome-like lipoplexes can efficiently deliver RNA to that is capable of systemically activating dendritic cells. In a phase I dose-escalation trial of this technology, melanoma patients treated at low doses displayed strong antigen-specific T-cell responses [90]. As cancer immunotherapy becomes standard-of-care for various cancers, similar exosome-based vaccination or therapeutic delivery strategies will become increasingly important. This therapeutic possibility has been explored in post-transplantation treatment, utilizing exosomes to prime mesenchymal stem cells for recovery [91].

## Conclusions

Exosomes, and the genetic material and proteins that they contain, have shown promise as useful indicators of tumor burden, prognosis, and perhaps even as therapeutic treatment, as they modulate so many aspects of heterotypic cell-cell interaction in the TME. These understandings can only expand through collaborative studies, drawing from rapid and continual advances in exosome isolation methods; sequencing depth, coverage, and speed; and the meticulous and precise process of characterizing and modeling signaling, interactions, and the dynamic interplay of the TME. As sensitivity of exosome isolation techniques improves, understanding the presence of specific exosomes in the tumor microenvironment and their genetic and protein cargo may allow them to serve as useful cell-free biomarkers in cancer prevention and targets for preempting or reversing cancer therapy resistance.

## Abbreviations

CAF: Cancer-associated fibroblast
EBV: Epstein-Barr virus
ECM: Extracellular matrix
ERCC: Extracellular RNA Communication Consortium
HCV: Hepatitis C virus
MDSC: Myeloid-derived suppressor cell
ncRNA: Non-coding RNA
NGS: Next-generation sequencing
NK: Natural killer
PRR: Pattern recognition receptor
rRNA: Ribosomal RNA
TLR: Toll-like receptor
TME: Tumor microenvironment
